# Constitutive Androstane Receptor Ligands Modulate the Anti-Tumor Efficacy of Paclitaxel in Non-Small Cell Lung Cancer Cells

**DOI:** 10.1371/journal.pone.0099484

**Published:** 2014-06-24

**Authors:** Heidge Fukumasu, Arina L. Rochetti, Pedro R. L. Pires, Edson R. Silva, Ligia G. Mesquita, Ricardo F. Strefezzi, Daniel D. De Carvalho, Maria L. Dagli

**Affiliations:** 1 Laboratory of Comparative and Translational Oncology, Department of Veterinary Medicine, School of Animal Science and Food Engineering, University of São Paulo, Pirassununga, Brazil; 2 Campbell Family Cancer Research Institute, Ontario Cancer Institute, Princess Margaret Cancer Centre, University Health Network, Toronto, Canada; 3 Department of Medical Biophysics, University of Toronto, Toronto, Canada; 4 Laboratory of Experimental and Comparative Oncology, Department of Pathology, School of Veterinary Medicine and Animal Science of the University of São Paulo, Sao Paulo, Brazil; Rajiv Gandhi Centre for Biotechnology, India

## Abstract

**Background:**

Lung tumors are the leading cause of cancer deaths worldwide and paclitaxel has proven to be useful for patients with lung cancer, however, acquired resistance is a major problem. To overcome this problem, one promising option is the use of Constitutive Androstane Receptor (CAR) ligands in combination with chemotherapeutics against cancer cells. Therefore, we wish to elucidate the effects of CAR ligands on the antineoplastic efficacy of paclitaxel in lung cancer cells.

**Methodology/Principal Findings:**

Our results from cell viability assays exposing CAR agonist or inverse-agonist to mouse and human lung cancer cells modulated the antineoplastic effect of paclitaxel. The CAR agonists increased the effect of Paclitaxel in 6 of 7 lung cancer cell lines, whereas the inverse-agonist had no effect on paclitaxel cytotoxicity. Interestingly, the mCAR agonist TCPOBOP enhanced the expression of two tumor suppressor genes, namely WT1 and MGMT, which were additively enhanced in cells treated with CAR agonist in combination with paclitaxel. Also, *in silico* analysis showed that both paclitaxel and CAR agonist TCPOBOP docked into the mCAR structure but not the inverse agonist androstenol. Paclitaxel per se increases the expression of CAR in cancer cells. At last, we analyzed the expression of CAR in two public independent studies from The Cancer Genome Atlas (TCGA) of Non Small Cell Lung Cancer (NSCLC). CAR is expressed in variable levels in NSCLC samples and no association with overall survival was noted.

**Conclusions/Significance:**

Taken together, our results demonstrated that CAR agonists modulate the antineoplastic efficacy of paclitaxel in mouse and human cancer cell lines. This effect was probably related by the enhanced expression of two tumor suppressor genes, viz. WT1 and MGMT. Most of NSCLC cases present CAR gene expression turning it possible to speculate the use of CAR modulation by ligands along with Paclitaxel in NSCLC therapy.

## Introduction

Lung tumors are the leading cause of cancer deaths worldwide, and they are responsible for estimated 1.2 million deaths per year [1]. In the last 30 years, several advances in lung cancer therapy have emerged with the improvement of immunotherapy, radiotherapy and chemotherapy, yet the gain in the survival time of lung cancer patients continue to be modest [2]. The treatment for lung cancer depends on the histologic type, the presence of metastasis and the patient's performance status. The most common treatment approaches include a combination of surgery (when tumors are resectable), radiotherapy and chemotherapy. Regarding the latter, the use of one or more cytotoxic drugs at the same time, such as taxanes, platinum compounds, and/or nucleoside analogs is most common. Generally, first-line chemotherapy for advanced non-small cell lung cancer (NSCLC) employs a protocol with a taxane (paclitaxel or docetaxel) associated with cisplatin or gemcitabine [3].

Cancers usually present as a heterogeneous population of malignant cells, with some that are drug-sensitive and some that are drug-resistant. Cytotoxic chemotherapy kills drug-sensitive cells, but does not affect drug-resistant cells that are generally in a dormant state [4]. As the tumor begins to grow again, chemotherapy often fails because the remaining tumor cells are primarily drug-resistant [5]. Paclitaxel, a widely used antineoplastic drug for lung cancer, is a tubulin-binding agent that blocks the progression of mitosis ultimately leading to cell death by apoptosis [3]. This taxane has proven to be a useful drug for patients with lung cancer; however, as with other chemotherapeutic drugs, acquired resistance by cancer cells is commonly observed.

Therefore, increasing the efficacy of paclitaxel is highly desirable. Chen *et al*. [6] considered one promising option involving the use of CAR (Constitutive Androstane Receptor, NR1I3) and PXR (pregnane-X receptor, NR1I2) ligands in combination with chemotherapeutics that activate PXR and CAR to overcome, or at least attenuate multi-drug resistance (MDR) in cancer cells. Interestingly, paclitaxel is a potent PXR activator and inducer of P-gp-mediated drug clearance [7]. In addition, several chemotherapeutic drugs are modulated or metabolized by the cytochrome P450 enzyme CYP3A4 [8], a known transcriptional target of activated PXR and CAR [9], [10].

CAR and PXR are steroid nuclear receptors known as master xenosensors [11] that are capable of recognizing structurally diverse compounds [12]. Both receptors, when activated by ligands, translocate to the nucleus and induce the transcription of several genes involved in drug metabolism and excretion, glucose and lipid metabolism and hormonal regulation [13], [14]. Recently, the importance of PXR in cancer pathogenesis and MDR of tumors has been a matter of debate, but no consensus on its specific role has been achieved so far [15], [16]. Similar to PXR, the role of CAR in cancer is also controversial. On in one hand, CAR was determined to be essential for liver tumor promotion by Phenobarbital [17], [18], and on the other hand CAR was shown to be a novel therapeutic target for brain and hematopoietic tumors [19], [20]. Therefore, our aim was to elucidate the importance of CAR modulation by selectively ligands and to determine the downstream effects on the antineoplastic efficacy of one of the most common used chemotherapeutic drugs for lung cancer.

## Material and Methods

### Reagents and cell lines

Paclitaxel, CITCO, TCPOBOP, androstenol and MTT were obtained from Sigma-Aldrich (St. Louis, MO, USA). Media and reagents for cell culture were acquired from Invitrogen (Carlsbad, CA, USA). Trizol, oligoDT primers, Superscript II enzyme and the Power SYBR Green mastermix were from Life Technologies (Grand Island, NY, USA). Other reagents were of analytical grade. The cell lines used in this experiment were the mouse cell line E9 [21] and the human cell lines A549, H2023, H460, H2030, H1792 and H23 [22]. All these cell lines were a gift from Dr. Lucy M. Anderson from the Laboratory of Comparative Carcinogenesis at the Frederick National Laboratory for Cancer Research (United States of America).

### Cell experiments with mouse and human lung cancer cell lines

Mouse lung cancer cell line E9 was originated from spontaneous transformation of immortalized non-neoplastic lung epithelial cells isolated from BALB/c mouse [23]. These cells were cultured in CMRL 1066 medium (Invitrogen, New York, NY), supplemented with 10% fetal calf serum (Invitrogen), 200 mM of L-Glutamine (Invitrogen) and antibiotic cocktail (100 units/mL penicillin and 100 mg/mL streptomycin; Invitrogen) in a humidified incubator at 37°C and 7%CO_2_. Human lung cancer cell lines were grown in RPMI 1640 (Invitrogen, New York, NY), with 10% fetal bovine serum (Invitrogen) plus 2% L-glutamine (Invitrogen) and 1% Pen-strep (Invitrogen) in a humidified incubator at 37°C and 5%CO_2_.

#### Determination of mCAR ligands effects on cell viability

E9 cells were seeded at 2000/well in 96 well plates (Corning, USA) containing 100 µl of supplemented media as described. After 24 h, media was discharged and changed with new media added with different concentrations of CAR agonist (TCPOBOP) or CAR inverse agonist (androstenol) from 10^−4^ µM to 10 µM. Forty-eight hours later, 11 µl of 3-(4,5-dimethylthiazol-2-yl)-2,5-diphenyl tetrazolium bromide (MTT - 5 mg/mL) was added to each well and formazan crystals were produced over a 2 h incubation period. Medium was removed from each well and 100 µl of 0.4N HCl in isopropilic alcohol were added to dissolve crystals. Optical density at 540 nm was measured in a Fluorstar Optima (BMG Labtech, Germany).

#### Determination of half maximal inhibitory concentration of Paclitaxel (IC50)

E9 cells were used with the same protocol described above with concentrations from 1 nM to 1600 nM of Paclitaxel.

#### Evaluation of the effects of mCAR ligands on paclitaxel cytotoxicity

E9 cells were used with the same protocol described above, where ligands were added simultaneously with the IC50 of Paclitaxel and cell viability was evaluated as described.

#### Determination of hCAR ligands effects on cell viability, calculation of the IC50 of Paclitaxel and co-exposure experiments

All these experiments using human cancer cell lines were performed as described for mouse E9 cancer cells with specific conditions for cell culture as described.

### Gene expression analysis of mCAR

E9 cells were seeded at 3.10^5^ cells/plate in T25 plates (Corning, USA) under the same conditions described above with the addition of TCPOBOP (10 µM), Androstenol (10 µM), TCPOBOP (10 µM) plus Paclitaxel (IC50), Androstenol (10 µM) plus Paclitaxel (IC50) or DMSO-only for control. Total RNA was extracted from five replicates of each treatment and controls with Trizol following the manufacturer's instructions. The RNA samples were then quantified (Biophotometer, Eppendorf, Germany) and the 260/280 ratio was observed. Only samples which presented 1.7–2.0 and demonstrated good quality (not degraded) after the electrophoresis analysis in an agarose gel (1.5%, Tris-buffered saline) were used. Thus, 1 µg of total RNA was reverse transcribed with oligoDT primers and superscript II into cDNA. All primers were designed with Primer-3 software [24] and were run in BLAST [25] to verify the absence of local alignments with DNA and other mouse RNA transcript sequences. Power SYBR Green was used for real-time PCR with primers for mCAR (NM_009803.5; F: 5′-GGGCCTCTTTGCTACAAGAT-3′; R: 5′-AGGTTTTTATGGAAGTGGAGGA-3′). The housekeeping gene used was the 18 s ribosomal RNA (NR_003278.3; F: 5′-CCTGCGGCTTAATTTGACTC-3′; R: 5′-CTGTCAATCCTGTCCGTGTC-3′). The reactions were carried out in an ABI Prism 7500 thermocycler (Life technologies, Grand Island, NY, USA) with the Power SYBR green Master Mix reagent and the analysis of relative gene expression data was performed according to Delta-Delta-CT method [26].

### PCR array RT^2^ profiler analysis

The RT^2^ Profiler Mouse Oncogenes and Tumor Suppressor genes PCR Array (PAMM-502Z, SABiosciences, USA), containing 84 genes that promote oncogenesis, plus housekeeping genes and controls, was used to analyze the effects of TCPOBOP plus Paclitaxel-related gene expression in E9 cells. Cells were seeded and treated as described above, total RNA was extracted with the RNeasy Mini kit (Qiagen, USA) and three replicates per treatment where pooled for analysis (experiment performed in duplicate). Pooled RNA was reverse-transcribed with the First Strand kit (SABiosciences), combined with the SYBR Green/ROX PCR master mix (SABiosciences), and added to each well of the RT2 Profiler PCR plate, containing the pre-dispensed gene-specific primer sets. The reaction was run on an ABI Prism 7500 thermocycler. Data analysis was based on the Ct method, with normalization to four different housekeeping genes. Fold changes of 2X (up or down-regulation) were considered for analysis.

### *In silico* analysis for docking of mCAR ligands and paclitaxel into the mCAR structure

Computational analysis was performed using the crystal structure of the CAR receptor co-crystallized with androstenol (pdb 1XNX) [27] and TCPOBOP (pdb 1XLS) [28]. Receptor target and docking ligands were prepared using Chimera [29]. The molecular surface of the target was generated based on the algorithm development [30]. Sphere generation was performed using the sphgen algorithm; the spheres were distributed with dock6 and selected using “spheres_selector”. Grid generation was achieved using Grid, which is distributed as an accessory to DOCK [31]. Flexible Dock was used to verify interactions between the target CAR receptor and chemicals [32]. Results obtained by docking were visualized and analyzed on Chimera version 1.4.1 (build 30365).

### cBioPortal analysis of The Cancer Genome Atlas data sets

cBioPortal, a tool developed by the Computations Biology Center at Sloan Kettering, was accessed at http://www.cbioportal.org/public-portal/ [33], [34]. Two data sets were used in this work: “Lung Adenocarcinoma (TCGA, in press)” with 230 cases and the “Lung Squamous Cell Carcinoma (TCGA, Provisional)” with 489 cases at the time of analysis, March 2014. Both studies were used to evaluate the presence of gene mutations and copy number alterations (CNA) illustrated by “oncoprints”, altered mRNA expression and/or DNA methylation and overall survival curves within these alterations. To determine which sample presented altered gene expression the Z-score was set to 1.96.

### Statistical analysis

Data are presented as mean ± standard deviation unless otherwise indicated. Graphpad Prism 5 for windows (Graphpad Software, USA) was used for all statistical analyses performed with nonparametric tests as Mann-Whitney and Spearman. Two-way ANOVA was used for comparisons between different ligands effects on cell viability. Overall survival from TCGA data were estimated with Kaplan-Meier curves and Logrank p-values by the cBioPortal for Cancer Genomics [33], [34]. Significant differences were considered when p<0.05.

## Results

### The CAR ligands are not cytotoxic to mouse or human lung cancer cells

**Figure 1:**
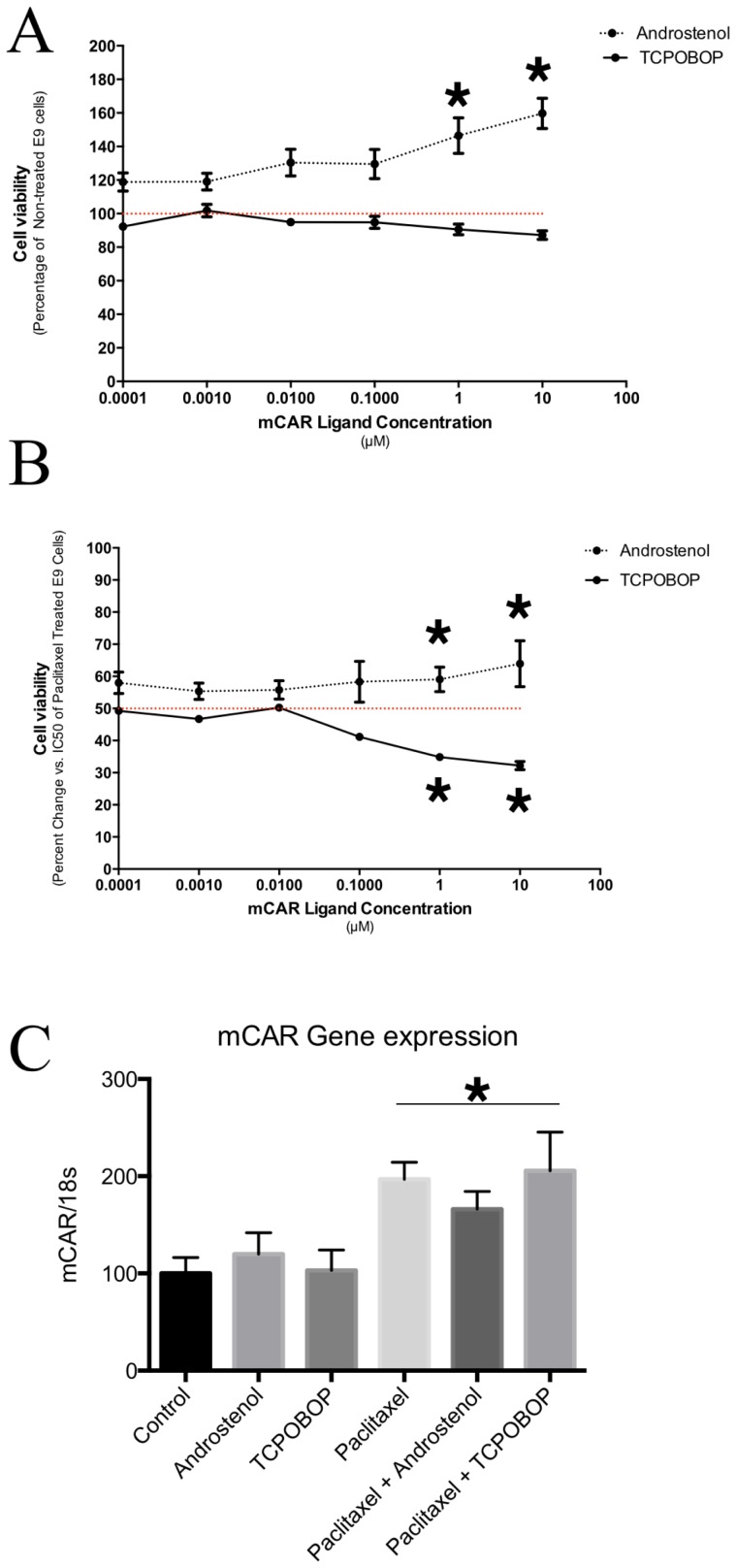
Effects of mCAR ligands alone or in combination with Paclitaxel in E9 mouse lung cancer cells. (A) Cell viability after 48 hours of different concentrations of the mCAR agonist TCPOBOP or the mCAR inverse-agonist androstenol. TCPOBOP has no effect on cell viability even at the highest concentration. On the other hand, androstenol increases the number of E9 cancer cells dose-dependently (* p<0.05 – Two way ANOVA followed by Tukey's multiple comparison test for treatment effect). (B) Effects of mCAR ligands on the anti-tumor efficacy of paclitaxel (IC50). TCPOBOP increases the anti-tumor efficacy of the of paclitaxel by significant decrease cell viability at 1–10 µM compared to only paclitaxel-treated cells (p<0.05 – Two way ANOVA followed by Tukey's multiple comparison test for treatment effect). The androstenol partially abolishes the anti-tumor efficacy of paclitaxel (p<0.05 – Two way ANOVA followed by Tukey's multiple comparison test for treatment effect). (C) Gene expression of mCAR in cells treated with ligands alone, paclitaxel alone, or in combination. Ligands did not alter mCAR gene expression. Note that all paclitaxel treated-groups presented increased mCAR gene expression compared to control group (* p<0.05 – One way ANOVA).

Initially, we evaluated the cytotoxic effects of mCAR ligands, including the agonist TCPOBOP and the inverse agonist androstenol, on E9 mouse lung cancer cells. Exposure to TCPOBOP for 48 h had no effect on E9 cell viability even at a high concentration (Fig.1). On the other hand, the inverse-agonist androstenol induced a dose-dependent increase in cell proliferation, with more than 60% cells than the control group in the high concentration (p<0.0001; Fig. 1). These results suggest that the use of mCAR agonists in mouse cancer cells might result in different effects on cell viability, even increasing cell proliferation as observed for androstenol.

Next, we performed similar experiments in six human lung cancer cell lines. We tested if the specific human CAR agonist, CITCO, and the inverse agonist androstenol, presented any cytotoxic effects in these cell lines. No effect was noted for CITCO or Androstenol even at the highest concentration tested (p>0.05 for all cell lines, Fig. S1).

### The CAR agonists TCPOBOP and CITCO enhance the antineoplastic efficacy of paclitaxel in mouse and human lung cancer cells

Our hypothesis was that CAR modulation by its ligands could alter the anti-tumor efficacy of paclitaxel, an antineoplastic agent commonly used for lung cancer chemotherapy in humans. First, we determined the concentration of paclitaxel that inhibited 50% of cell viability for each cell line (IC50, Table 1). Using this concentration, we next evaluated the effects of CAR agonists on the anti-tumor effect of Paclitaxel by exposing cancer cells to paclitaxel plus different concentrations of a CAR agonist or inverse-agonist.

**Table 1:** IC50 of Paclitaxel in mouse and human lung cancer cell lines.

*Cell line*	*Specie*	*Histopathology*	*Derived from:*	*Paclitaxel IC50 (µM)*
E9	Mouse	Adenocarcinoma	Primary site	0.228
A549	Human	Carcinoma	Primary site	8.194
H23	Human	NSCLC	Primary site	2.136
H460	Human	Carcinoma	Pleural effusion	1.138
H1792	Human	Adenocarcinoma	derived from metastatic site: pleural effusion	8.087
H2023	Human	NSCLC	derived from metastatic site	4.175
H2030	Human	NSCLC	derived from metastatic site: lymph node	2.474

NSCLC – Non-Small Cell Lung Cancer.

When we co-exposed mouse lung cancer cells to different concentrations of TCPOBOP plus paclitaxel at the inhibitory concentration (IC50), the CAR agonist improved paclitaxel anti-tumor efficacy dose-dependently, reducing cell viability by almost 40% when compared to that in cells treated with Paclitaxel alone (p<0.05; Fig. 1). On the other hand, the inverse agonist androstenol reduced the cytotoxic effects of paclitaxel in E9 cells (p<0.05; Fig. 1). These results indicate that specific modulation of CAR by the agonist TCPOBOP improves the anti-tumor efficacy of paclitaxel in E9 cancer cells.

**Figure 2:**
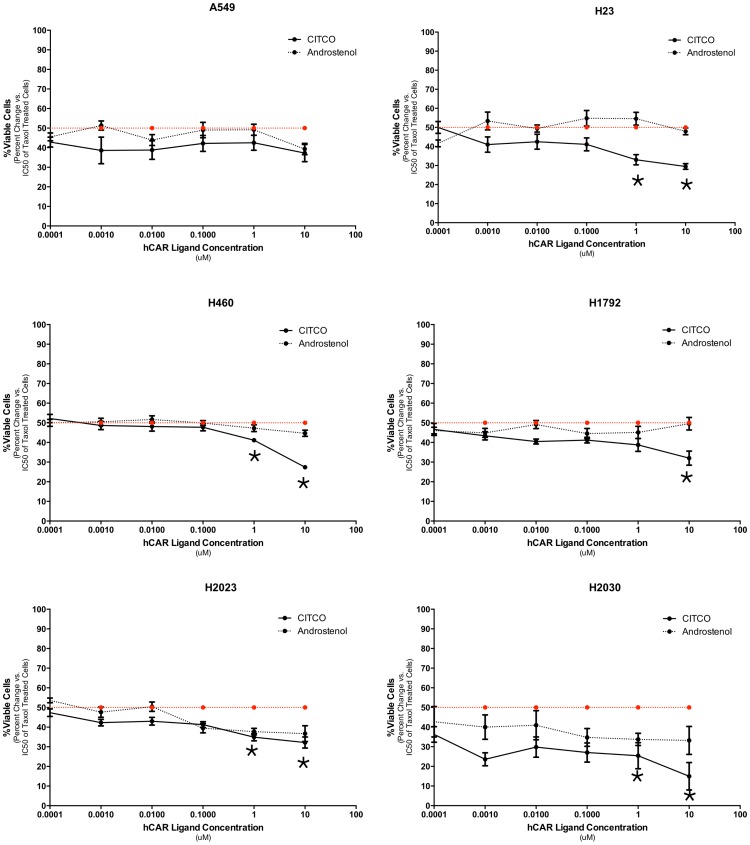
Effects of hCAR ligands on the anti-tumor efficacy of paclitaxel in six different human lung cancer cells. Cell viability after 48-agonist Androstenol in combination with Paclitaxel. CITCO enhances paclitaxel cytotoxicity significantly in five of six cell lines (* p<0.05 – Two way ANOVA followed by Tukey's multiple comparison test for treatment effect). On the other hand, androstenol in combination with paclitaxel has no effect in comparison to paclitaxel-treated cells.

We performed this experiment in six human lung cancer cell lines where the co-exposure of human CAR ligands was evaluated for its effect on the citotoxicity of paclitaxel. The CAR agonist CITCO significantly enhanced paclitaxel efficacy in five of the six human lung cancer cells tested (p<0.05; Fig. 2). On the other hand, the CAR inverse-agonist androstenol resulted in no significant differences in any cell lines (p>0.05; Fig. 2). These results demonstrate that CAR agonists enhance the antineoplastic effect of paclitaxel in the majority of lung cancer cell lines (mouse and humans), making them an interesting focus for further characterization.

### Paclitaxel enhances mCAR expression

We next evaluated wheter paclitaxel altered mCAR gene expression. To this end, we exposed E9 cells to the following different treatments: 10 µM of TCPOBOP, 10 µM of androstenol, the IC50 of Paclitaxel, or a combination of these. Our results showed that a single treatment with TCPOBOP or androstenol had no effect on mCAR mRNA expression (Fig. 1). However, when cells were treated with paclitaxel alone or in combination with TCPOBOP or androstenol, an increase in mCAR expression was seen independent of the ligand (p = 0.0102; Fig. 1).

### TCPOBOP and paclitaxel alter tumor suppressor and oncogene expression levels

Further experiments were performed only with the mouse lung cancer cell line E9. We evaluated the effects of TCPOBOP and paclitaxel on the gene expression profile of 84 oncogenes and tumor suppressor genes. Initially, five genes were cut-off from analysis because they presented CT values greater than 35 and/or the presence of more than one PCR product was detected. From the 79 remaining genes, paclitaxel treatment altered the expression of 10 genes, with fold changes of more than 2 (Table 2). According to the functional gene groupings from the Rt2 Profiler PCR array, most of genes up-regulated paclitaxel were tumor suppressor genes, genes related to apoptosis, oncogenes or genes that present properties of oncogenes and tumor suppressor genes (Table 2).

**Table 2:** Altered gene expression by Paclitaxel, TCPOBOP and their combination in E9 cells.

Gene/Fold Change	Paclitaxel/Control	Combination/Control	TCPOBOP/Control	Function
*Up-regulated genes*				
MGMT	**54,52**	**86,2**	**3,19**	TSG
TO73	**4,14**	**3,17**	1,13	TSG
RET	**4,04**	**3,43**	−1,08	TSG&ONCO
ROS1	**3,89**	**6,90**	1,52	ONCO
RASSF1	**2,93**	**2,72**	1,10	TSG
WT1	**2,92**	**4,19**	**2,81**	TSG
MOS	**2,74**	1,57	−1,16	ONCO
ELK1	**2,26**	**2,11**	1,33	ONCO
CASP8	**2,26**	**2,24**	1,21	APOPT
NFKBIA	**2,03**	1,63	−1,41	ONCO
MYCN	1,56	**2,25**	1,85	TSG&ONC
*Down-regulated genes*				
ZHX2	−1,69	**−2,09**	−1,29	ONCO
ESR1	−1,28	**−2,16**	−1,01	TSG&ONC

Red values means increased gene expression by ≥2 fold. Blue values means decreased gene expression by ≤ 2 fold. TSG – Tumor suppressor gene; ONCO - Oncogene; APOPT – apoptosis-related gene

The exposure of E9 cells to the mouse CAR ligand TCPOBOP increased the expression of two tumor suppressor genes: MGMT and WT1 (Table 2). Interestingly, when we evaluated the gene expression of cells treated with TCPOBOP plus paclitaxel (with the same concentration that increases paclitaxel cytotoxicity), the expression of these two genes was enhanced further than when only paclitaxel or TCPOBOP were administered, suggesting an additive effect on the gene expression of these genes. In addition, the combination of TCPOBOP plus paclitaxel resulted in the downregulation of two oncogenes, ZHX2 and ESR1 (Table 2). Moreover, it seems that the combined exposure of E9 cells to TCPOBOP and paclitaxel enhanced the paclitaxel-induced gene expression signature (Table 2). Taken together, these results are in accordance with the above described and explain the modulatory properties of the mCAR agonist TCPOBOP on the anti-tumor effect of paclitaxel in mouse lung cancer cells.

### In silico analysis shows that Paclitaxel and TCPOBOP fit into the mCAR structure

**Figure 3:**
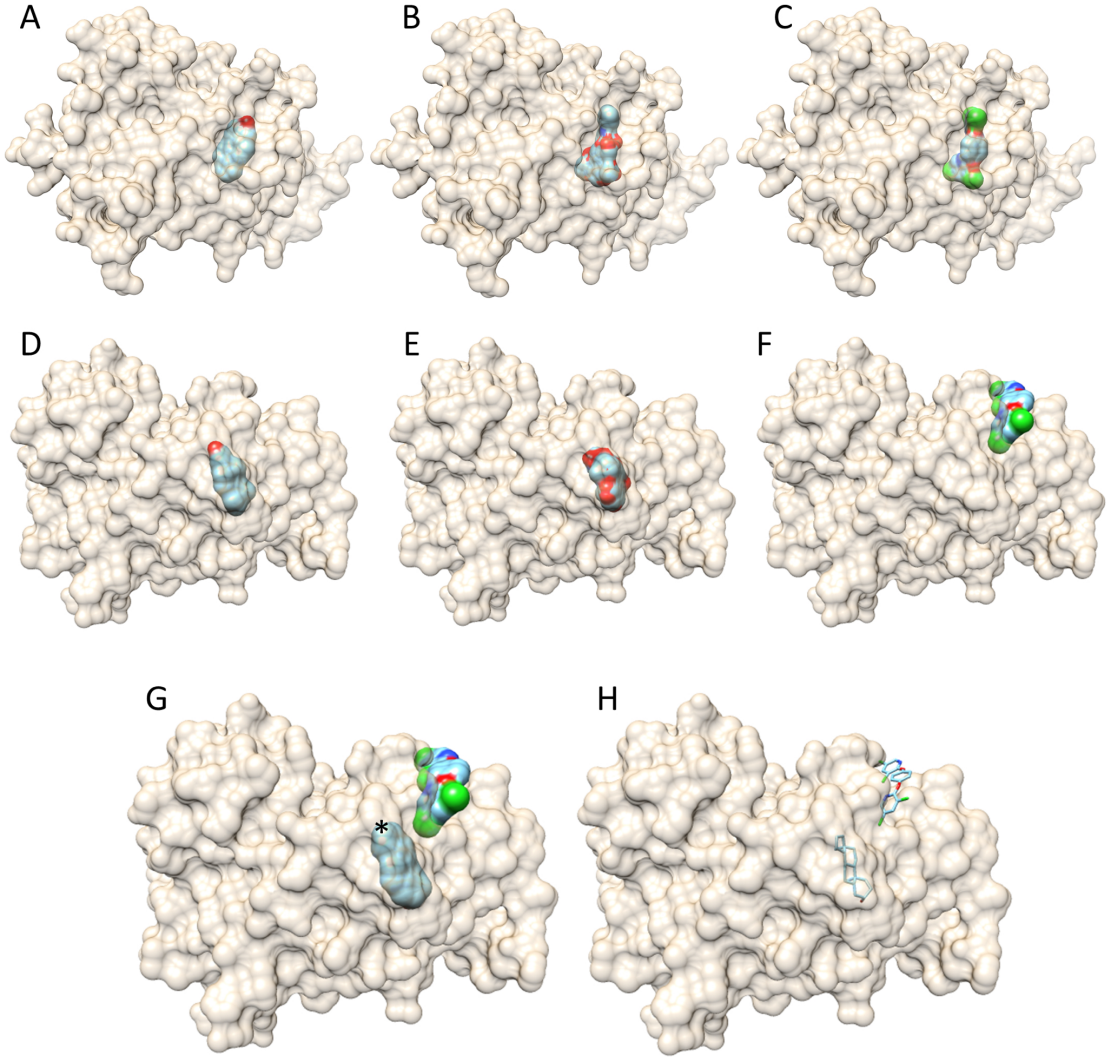
Docking using CAR crystal structure with ligand (PDB-1XLS) TCPOBOP (A, B, and C) and with ligand (PDB-1XNX) androstenol (D-H). A, D-Androstenol; B, E-Paclitaxel; C,F-TCPOBOP; G and H *androstenol and TCPOBOP superposition: G- surface view and H- wire view of ligands. Note that paclitaxel docked in both mCAR structures.

Alignment of 1XLS and 1XNX structure showed that the positioning of the agonist TCPOBOP and the inverse-agonist androstenol into the mCAR structure are distinct (Fig. 3). The docking of TCPOBOP was performed as a control, and it showed a good superposition of TCPOBOP in the crystal structure of 1XLS (Fig. 3). The docking using the 1XLS structure revealed that the inverse-agonist androstenol could not bind at the position of the agonist TCPOBOP; androstenol showed a positive energy grid (51.82) that is unfavorable to bind inside the receptor. The energy grid of TCPOBOP and paclitaxel are respectively −49.34 and −125.53. On the other hand, docking using the 1XNX structure to guide the best energy to the receptor-ligand interaction pointed to a possible second site in the CAR receptor for TCPOBOP binding (Fig. 3) with a favorable energy grid (−32.01), similar to the one obtained for the androstenol (−40.44). Paclitaxel also showed the most favorable energy and closed binding values of bind in both structures where TCPOBOP or androstenol were used as a docking guide ligand (Table 3), which indicates that paclitaxel might also be a mCAR ligand.

**Table 3:** Energy grid of mCAR ligands and paclitaxel docking against XLS and XNX structures.

	Energy Grid	
	XLS	XNX
TCPOBOP	−49,34	−32,01
Androstenol	51,82	−40,44
Paclitaxel	−125,53	−121,18

### Human CAR characterization in Non-small Cell Lung Cancer samples from two independent studies

**Figure 4:**
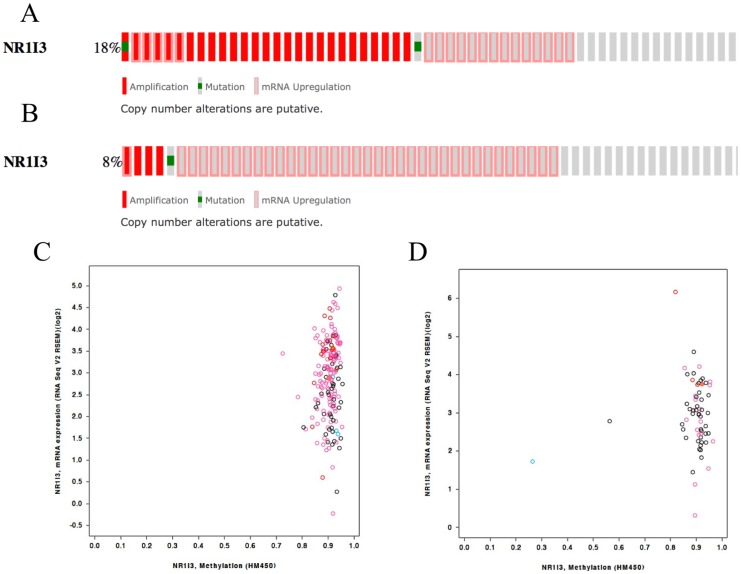
hCAR characterization in two NSCLC independent studies. (A) Genetic alterations and frequency of hCAR in the Lung Adenocarcinoma study available from TCGA. (B) Same data from the Lung Squamous Cell Carcinoma study available from TCGA. (C) DNA methylation vs gene expression of hCAR from Lung Adenocarcinoma samples from TCGA. (D) DNA methylation vs gene expression of hCAR from Lung Squamous Cell Carcinoma samples from TCGA. Both datasets presented highly DNA methylation and variable levels of mRNA expression of NR1I3.

We characterized the status of hCAR in NLSLC from two studies with publicly available data through TCGA. In the adenocarcinoma study, from 230 samples, 17.8% of the cases (41/230) presented genetic alterations of hCAR, including Copy Number Alterations (CNA), mutations or altered gene expression (Fig. 4). These alterations were not associated with overall survival (OS, p = 0.40, Fig. S2). The majority of the samples from this study presented with increased hCAR methylation according to the HM450 analysis (Fig. 4). Corroborating this data, only 8% of the cases (19/230) presented with upregulation of hCAR mRNA.

Next, we used information from another study from TCGA on lung squamous cell carcinoma (LSCC, TCGA, provisional data - 04/26/2014). The frequency of hCAR alterations including mutations, CNAs or altered gene expression (Fig. 4), was 8.2% (40/489) which was not associated with OS (p = 0.81, Fig. S1). Similar to the adenocarcinoma study, the majority of the samples presented with increased DNA methylation of hCAR (Fig. 4). This may explain why only 7.4% of all cases presented with upregulation of hCAR mRNA (36/489).

Based on the results from both studies, it is possible that the majority of the samples from lung adenocarcinoma and LSCC patients present no alterations in hCAR expression in relation to the paired, non-cancerous tissue, which could be explained by the high levels of methylation found in both studies.

## Discussion

Lung cancer continues to be a major problem for health-care systems worldwide, as most cases involve patients with comprised lung function, metastasis, multi-drug resistance and poor outcomes. The survival of lung cancer patients after tumor detection is typically less than 5% at five years [35]. Recently, evidence has started to show that CAR might have a role in cancer therapy [19], [20]. Here we demonstrate that specific CAR modulation by agonists, but not by inverse-agonists, increased the anti-tumor efficacy of paclitaxel in both murine and human lung cancer cells. This effect was accompanied by the potentiation of a paclitaxel-induced gene expression signature when using a combination of paclitaxel and TCPOBOP. In addition, a single exposure of cancer cells to TCPOBOP increased the expression of two tumor suppressor genes (WT1 and MGMT), which could corroborate the improved efficacy of paclitaxel in cancer cell lines. Paclitaxel treatment increased CAR gene expression and docked into the molecular structure of CAR *in silico*. Finally, we analyzed the profile of hCAR in two TCGA studies and noted that CAR could be an interesting target for NSCLC patients receiving paclitaxel treatment since hCAR is expressed in tumor samples and has no association with OS.

Chen *et al.* recently considered CAR ligands to be a promising option for combined chemotherapy, with the aim of overcoming MDR in cancer cells [6]. Our results support this proposition since the CAR ligands did not cause cytotoxic effects per se in cancer cells; however, when CAR agonists were used in combination with paclitaxel, an interesting modulatory effect emerged. This enhanced cytotoxic effect was also seen in five of six different human cancer cell lines since CITCO, the hCAR agonist, showed a similar effect on paclitaxel efficacy. Therefore, we support the hypothesis of Chen *et al.* that CAR modulation might indeed be important for cancer chemotherapy [6].

The exact role of CAR in carcinogenesis and cancer therapy is still a matter of debate. On one side, CAR is essential for liver tumor promotion by phenobarbital in mice [18] and it regulates tumorigenesis in response to xenobiotic stress [36]. On the other side, Wang et al. described CAR as a novel therapeutic target that facilitates cyclophosphamide (CPA)–based treatment of hematopoietic malignancies [20], and where they concluded that CAR activation facilitates CPA-based chemotherapy by selectively promoting its bioactivation. In another study, CITCO was shown to target brain tumor stem cells, inhibiting their growth and expansion [19]. Both papers suggest the use of CAR agonists for the treatment of blood malignancies and brain cancers, respectively.

As previously stated, paclitaxel induces apoptosis in proliferating cells. Here, paclitaxel induced cell death in all mouse and human cancer cell lines, with unique IC50 values for each one of them. Even after considering that these cells present differences in their resistance to Paclitaxel (≈3600x), the CAR agonists improved the efficacy of paclitaxel in almost all cancer cell lines (6/7). On the other hand, the inverse-agonist Androstenol did not result in any effect on the cytotoxicity of paclitaxel in the majority of cells, and it attenuated the cytotoxic of paclitaxel in one cell line. These results lead to the conclusion that only CAR agonists should be considered to improve NSCLC cancer therapy.

The effects of CAR agonists on the anti-tumor efficacy of paclitaxel were similar in both mouse and human cells. Thus, we focused our experiments on the mouse model for two reasons: we have more experience with this model [37], [38] and all of the techniques used in the mouse model were routine in our lab. Furthermore, the use of mouse lung epithelial cancer cell lines has been established for more than 30 years now and has presented similar results to those obtained with human cell lines [23]. Thus, we also demonstrated in this *in vitro* mouse model that paclitaxel increases CAR gene expression at the IC50, independent of its association with CAR ligands. One could expect that mCAR ligands should modulate mCAR gene expression, but our results did not show this effect. CAR activation by specific ligands did not always alter CAR gene expression or protein levels, but it can still result in an increase in its transcriptional activity.

CAR expression analysis in mouse cancer cells treated with CAR ligands alone, paclitaxel alone, or a combination of both, revealed that Paclitaxel increased the gene expression of CAR independent of its association with TCPOBOP or androstenol. Accordingly Agreeing with this result, *in silico* docking analysis of TCPOBOP, Androstenol and Paclitaxel into CAR protein structure demonstrated that Paclitaxel fits into the ligand-binding pocket of mCAR receptor, which could increase CAR expression by positive feedback. No effect on CAR gene expression was noted after CAR ligands exposure, a fact that corroborates with the absence of cytotoxicity by these ligands.

Another interesting result supporting the modulatory effect of CAR agonists on paclitaxel efficacy was their potentiation of paclitaxel's gene expression signature, increasing the expression of some tumor suppressor genes and decreasing the expression of two oncogenes. Interestingly, treatment with TCPOBOP alone also induced the expression of two tumor suppressor genes MGMT and WT1. MGMT promoter methylation is a stronger prognostic factor than age, stage, and tumor grade for gliomas [39]. Furthermore, a higher frequency of this epigenetic feature was found in NSCLC indicating that it might be a common event during NSCLC carcinogenesis [40]. In fact, there is a strong association between MGMT promoter methylation and NSCLC as demonstrated by a meta-analysis study [41]. In our experiment, MGMT was the most altered gene following Paclitaxel treatment. Interesting, the exposure of cancer cells to a combination of Paclitaxel and TCPOBOP increase even further, indicating that TCPOBOP potentiates MGMT gene expression, probably because TCPOBOP triplicates MGMT gene expression alone. However, when we analyzed TCGA data from the two studies of NSCLC, only a small proportion of cases presented with MGMT DNA methylation (data not shown). Therefore, further studies on the role of MGMT methylation in NSCLC should establish its importance for clinical practice.

The other tumor suppressor gene induced by TCPOBOP in cancer cells, WT1, was previously shown to be expressed at lower levels in fatal cases of NSCLC than in survival cases [42]. These authors demonstrated that OS and disease-free survival of the high WT1 expression group were longer compared to those of the lower expression group. Finally, they concluded that low WT1 expression predicted poor prognosis in patients with NSCLC. On the other hand, two recently published papers from the same group determined that WT1 promotes the invasion of NSCLC via the suppression of E-cadherin, and it increases cell proliferation in NSCLC cell lines through upregulation of cyclin D1 and pRb [43], [44]. Therefore, the exact role of WT1 in NSCLC is a current matter of debate, and more prospective studies are required to confirm its importance. In our work, the exposure of cancer cells to a combination of paclitaxel and TCPOBOP increased WT1 gene expression compared to that with treatment with paclitaxel or TCPOBOP alone, making combination treatment of CAR agonists with paclitaxel a potentially interesting option for NSCLC therapy.

We evaluated more than 500 cases of NSCLC from two TCGA studies and the analysis showed that in the majority of cases, hCAR DNA is highly methylated; these results provide a possible explanation for 92% of the cases having very similar gene expression profiles. Thus, it is important to note that hCAR expression in NSCLC cases is not associated with OS, indicating that CAR modulation might be a good option for future experiments that seek to enhance paclitaxel's anti-tumor efficacy. We are aware that here we used *in vitro* data coupled with *in silico* analysis of two studies from TCGA and that future experiments should address the effect of this combination *in vivo*.

To our knowledge, this is the first study to demonstrate that CAR agonists modulate the antineoplastic effects of paclitaxel in different NSCLC cell lines. The enhancement of the paclitaxel-induced gene expression signature though co-exposure with TCPOBOP corroborates our conclusion. Interestingly, is the fact that the mCAR agonist TCPOBOP induced the expression of two tumor suppressor genes that are likely related to the enhanced antineoplastic effect of paclitaxel. In addition, the result that hCAR is expressed at variable levels in NSCLC samples suggests the possibility of using CAR agonists in combination with paclitaxel in the future.

## Supporting Information

Figure S1 **Effects of hCAR ligands in six different human lung cancer cells.** Cell viability after 48 hours of different concentrations of the hCAR agonist CITCO or the hCAR inverse-agonist androstenol. No effects were noted in all the six human cancer cell lines.
(TIF)

Figure S2 **Lack of association of NR1I3 alterations with overall survival from two independent studies of NSCLC.** Overall survival Kaplan-Meier estimates for cases with (red line) or without (blue line) NR1I3 alterations that include mutations, CNAs and altered gene expression. (A) Lung Adenocarcinoma cases. Logrank test p-value = 0.40. (B) Lung Squamous Cell Carcinoma cases. Logrank test p-value = 0.81.
(TIF)
