# Supplementary figures and images for: Constitutive Androstane Receptor Ligands Modulate the Anti-Tumor Efficacy of Paclitaxel in Non-Small Cell Lung Cancer Cells
Source: PLoS One. 2014 Jun 24;9(6):e99484. doi: 10.1371/journal.pone.0099484 (PMC4069004; doi:10.1371/journal.pone.0099484)

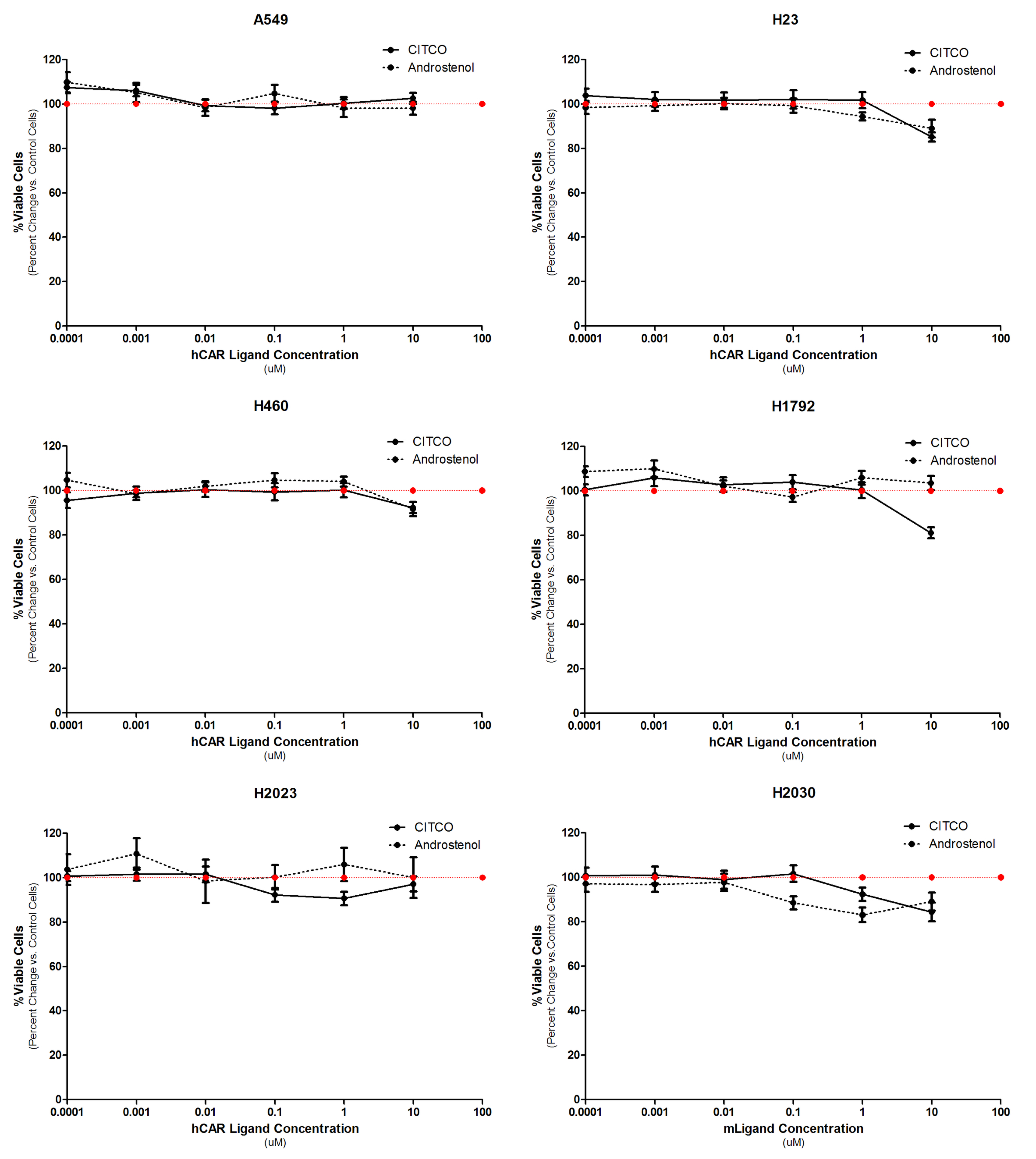

Supplement: Figure S1 — Effects of hCAR ligands in six different human lung cancer cells. Cell viability after 48 hours of different concentrations of the hCAR agonist CITCO or the hCAR inverse-agonist androstenol. No effects were noted in all the six human cancer cell lines. (TIF) [file pone.0099484.s001.tif]

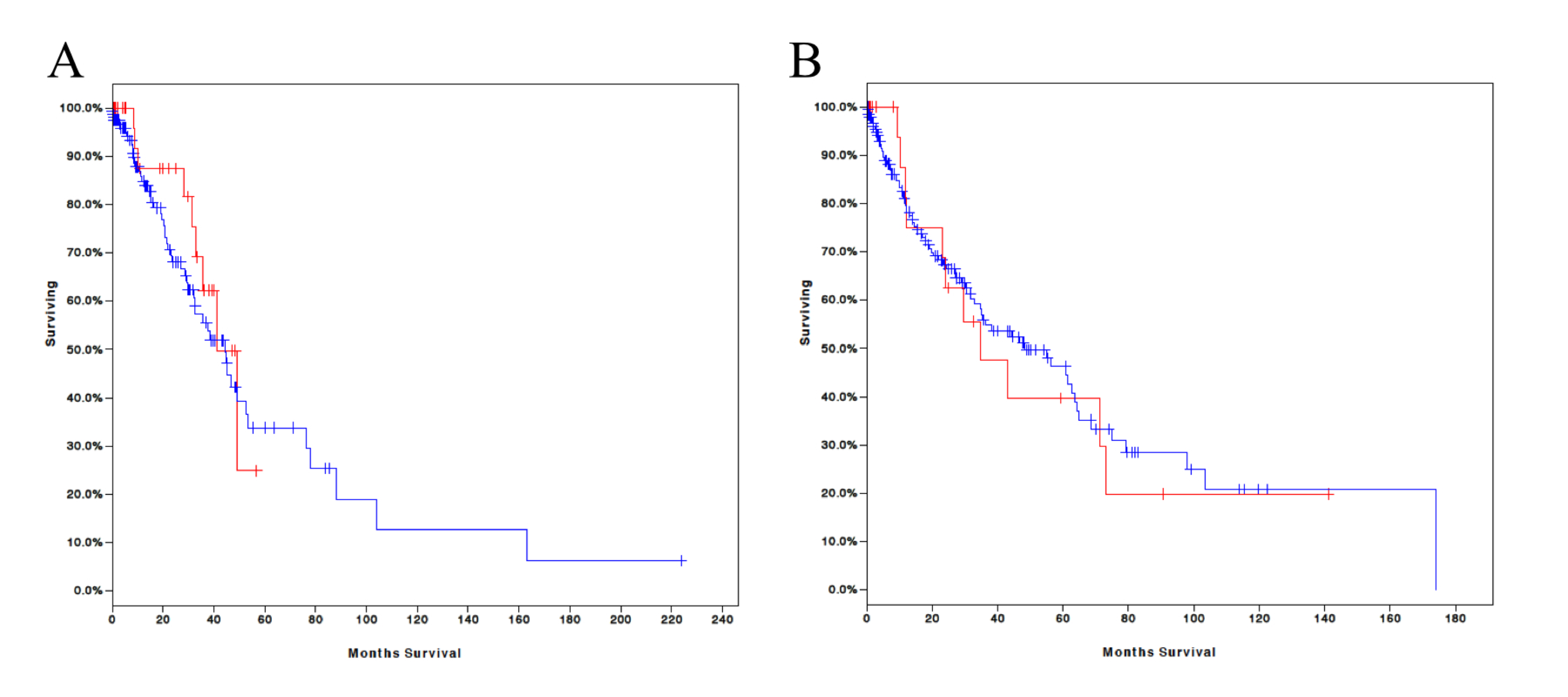

Supplement: Figure S2 — Lack of association of NR1I3 alterations with overall survival from two independent studies of NSCLC. Overall survival Kaplan-Meier estimates for cases with (red line) or without (blue line) NR1I3 alterations that include mutations, CNAs and altered gene expression. (A) Lung Adenocarcinoma cases. Logrank test p-value = 0.40. (B) Lung Squamous Cell Carcinoma cases. Logrank test p-value = 0.81. (TIF) [file pone.0099484.s002.tif]
